
JOURNAL INFORMATION
==============================
NLM Title Abbreviation: Wirtschaftsdienst
Journal ID: Wirtschaftsdienst
NLM Journal ID: 101086612

Wirtschaftsdienst (Hamburg, Germany : 1949)

ISSN: 0043-6275

ARTICLE INFORMATION
==============================
PMCID: PMC8598269
PMID: 34812207
DOI: 10.1007/s10273-021-3043-x
Article ID: 3043
Article version: 1
Subjects: Zeitgespräch

Globalisierung trifft Geoökonomie

Görg Holger holger.goerg@ifw-kiel.de
1
Kamin Katrin katrin.kamin@ifw-kiel.de
2
1 grid.462465.7 0000 0004 0493 2817 Forschungsgruppe “Global Division of Labour”, Institut für Weltwirtschaft Kiel, Kiellinie 66, 24105 Kiel, Deutschland
2 grid.462465.7 0000 0004 0493 2817 Trade Policy Task Force, Institut für Weltwirtschaft Kiel, Kiellinie 66, 24105 Kiel, Deutschland
Electronic publication date: 2021 Nov 18
Print publication date: 2021
Volume: 101
Issue: 11
First page: 854
Last page: 857
Copyright: © Der/die Autor:in 2021
License: Open Access: Dieser Artikel wird unter der Creative Commons Namensnennung 4.0 International Lizenz veröffentlicht (https://creativecommons.org/licenses/by/4.0/deed.de).
Open Access wird durch die ZBW — Leibniz-Informationszentrum Wirtschaft gefördert.
License URL: https://creativecommons.org/licenses/by/4.0/

issue-copyright-statement: © The Author(s) 2021

==============================

Ein rasanter Trend zur Globalisierung hat die letzten Jahrzehnte des 20. Jahrhunderts maßgeblich geprägt. Insbesondere seit 1990 waren konstant hohe Wachstumsraten des Welthandels zu beobachten, die um einiges höher waren als das generelle Wachstum der Weltwirtschaft (vgl. Abbildung 1). Nach kleineren Einbrüchen der Wachstumsraten in den späten 1990er und frühen 2000er Jahren, hat jedoch die weltweite Finanzkrise in 2008/2009 Sand ins Getriebe der Globalisierung gestreut und diesen Trend jäh unterbrochen. Das negative Wachstum des Handelsvolumens, welches in 2009 zu beobachten war, wurde jedoch durch positive Rekordraten 2010 und 2011 rasch wieder wettgemacht. Nach einer kurzen Erholungsphase kam dann die nächste Ladung Sand in Gestalt von protektionistischen Maßnahmen in mehreren Ländern. Dies hat dazu geführt, dass spätestens seit Beginn der US-Präsidentschaft von Donald Trump die Globalisierung von großen Verwerfungen geprägt ist.

Präsident Trumps America-First-Politik – die auch unter Präsident Biden noch spürbar ist – ist ein Zeichen eines neuen starken Trends: das stärkere Zurückgreifen von Regierungen auf geoökonomische Mittel zur Erreichung von außenpolitischen aber auch ökonomischen Zielen. Dieser Trend wird vermutlich die internationale Handelspolitik, aber auch andere Politikbereiche, im 21. Jahrhundert maßgeblich prägen. Geoökonomische Instrumente umfassen beispielsweise Sanktionen und Exportbeschränkungen, aber auch die strategische Beschränkung oder Öffnung des Zugangs zu Anleihen, Finanztransaktionen oder Wirtschaftshilfe. Allesamt im Grunde protektionistische Maßnahmen, die den internationalen Austausch von Gütern, Kapital, Menschen oder Technologie behindern.

So haben die USA Handelsbarrieren in Form von Zöllen gegenüber China und anderen Handelspartnern ausgebaut. Dies eskalierte in einem Handelskrieg zwischen den USA und China, der selbst nach Ende der Präsidentschaft Trump noch anhält. Dies hat massive Auswirkungen nicht nur auf diese zwei Länder, sondern auch auf deren Handelspartner. Für die USA und China beispielsweise hatte die stufenweise Anhebung der gegenseitigen Zölle bis hin zu durchschnittlich mehr als 20 % insbesondere negative Auswirkungen auf die reale Produktion, aber ebenso auf die Wohlfahrt der beiden Länder (Mao und Görg, 2020; Felbermayr et al., 2021).

Zur gleichen Zeit wurden die Prinzipien des internationalen Handels und die Rolle der WTO durch diese und andere Auseinandersetzungen unterminiert. So stehen die im Wirtschafts- und Handelsabkommen (ETA) zwischen den USA und China vereinbarten Zielgrößen für den bilateralen Handel im Widerspruch zu Artikel 1 des GATT, welcher die WTO-Mitglieder zu einer diskriminierungsfreien Handelspolitik verpflichtet (Chowdhry und Felbermayr, 2020). Darüber hinaus hat die Blockadepolitik der USA im Hinblick auf den Appellate Body der WTO anderen Mitgliedstaaten verwehrt, sich gegen mögliche Rechtsverletzungen auf WTO-Ebene zu wehren. Zwar arbeiten die EU und China an Reformen, um solche Blockaden zu verhindern (Garcia-Herrero et al., 2020), dennoch hat der Multilateralismus dadurch großen Schaden davongetragen. Für die WTO, die sich zwar bereits seit dem Scheitern der Doha-Runde in der Krise befindet, haben diese Friktionen die Schwächen der Institution noch einmal deutlich hervorgehoben.

Währenddessen hat China seinerseits zunehmend in geoökonomische Megaprojekte wie die Neue Seidenstraße investiert und auch seine strategischen Investitionen in Afrika massiv ausgebaut. In dem Bestreben, Chinas Ausbau seiner geopolitischen Einflusssphäre etwas entgegenzusetzen, haben die G7 im Juni 2021 die Initiative Build Back Better World (B3W) gestartet, welche Privatinvestitionen in Infrastrukturprojekte in Entwicklungsländern fördern soll. Aber während die Transition von ökonomisch getriebener Außenwirtschaftspolitik hin zu einer stärker außenpolitisch und geopolitisch motivierten Außenwirtschaftspolitik in den USA erst in den vergan-

Abbildung 1
**Entwicklung des Welthandels und Welt-BIP**
prozentuales Wachstum

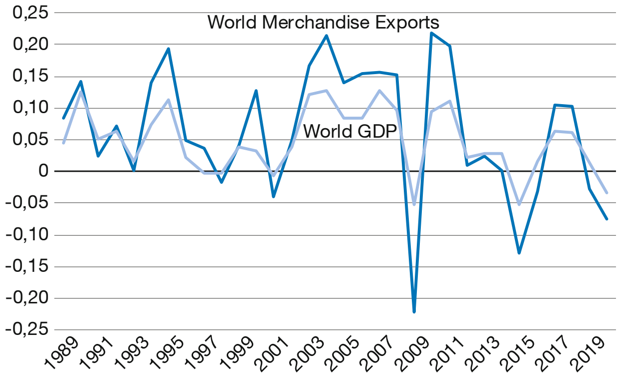

Quelle: eigene Berechnung basierend auf Daten der Weltbank (World Development Indicators).

genen Jahren vollzogen wurde und in der EU erst in den kommenden Jahren zu erwarten ist (Pisani-Ferry, 2021), verwendet China bereits versiert geoökonomische Instrumente. So ist die Investitionspolitik Chinas an politische Bedingungen geknüpft und die Verträge sind meist undurchsichtig. Zudem schließen sie eine Umschuldung oftmals explizit aus (Gelpern et al., 2021).

Zudem ist weltweit zunehmend die Anwendung eines weiteren geoökonomischen Druckmittels zu beobachten: Sanktionen. Die Zahl der verhängten Sanktionen ist von etwa 20 im Jahr 1950 auf etwa 250 im Jahr 2019 gestiegen (Felbermayr et al., 2020). Diese werden im Gegensatz zu den breit angelegten Sanktionsregimen der 1970er- und 1980er-Jahre etwa gegen Kuba oder den Iran, unter denen die Bevölkerung massiv litt, immer gezielter auf die Sektoren Finanzen, Reisen, militärische Unterstützung und Handel ausgerichtet.

Die COVID-19-Pandemie und der in vielen Ländern damit einhergehende Fokus auf nationale Interessen haben diese protektionistischen Bestrebungen darüber hinaus noch verschärft. Zum einen wurden für in der Pandemie benötigte medizinische Produkte Exportbeschränkungen erlassen, und es entbrannte ein Wettlauf um Impfstoffe (Fuchs et al., 2020). Zum anderen haben diese Exportbeschränkungen gepaart mit oben genannten geoökonomischen Mitteln den Druck für manche Länder wie beispielsweise den Iran drastisch erhöht (Chowdhry et al., 2020a).

Diese protektionistischen Maßnahmen, egal welche Gestalt sie annehmen, wirken sich negativ auf den Handel und andere Aspekte der Globalisierung aus. Handelsbarrieren erhöhen die Kosten importierter Produkte, was die Nachfrage reduziert. Negative Schlagzeilen über bestimmte Länder, oder gar Boykottaufrufe können ebenfalls dazu beitragen, dass Konsument:innen Produkte bestimmter Herkunftsländer scheuen und damit den Handel reduzieren. Darüber hinaus denken mittlerweile viele Unternehmen darüber nach, globale Lieferketten zu verkürzen und die Produktion „nach Hause" zu holen. Laut einer Studie von Flach et al. (2021) geben etwa 10 % der deutschen Unternehmen in einer Befragung an, dass sie beabsichtigen, in Zukunft mehr Vorprodukte und Materialien aus dem heimischen oder EU-Raum zu beziehen und damit die Lieferkette (sprich Handelsbeziehungen) zu verkürzen.

Da die Nutzung geoökonomischer Instrumente nichts Neues ist, stellt sich die Frage, was die Geoökonomie gerade jetzt zu einem so wichtigen Trend macht. Der Rückzug der USA aus ihrer hegemonialen Rolle sowie der Aufstieg Chinas und anderer Global Player haben die politische Weltordnung verändert. Diese hat sich weg von einer regelbasierten hin zu einer machtbasierten Ordnung verschoben. Defizite und Schwächen in den internationalen Institutionen, insbesondere in der WTO, haben dies weiter befeuert. Darüber hinaus hat die Globalisierung die Welt enger vernetzt und so auch mehr Abhängigkeiten und Verwundbarkeiten geschaffen, welche nun genutzt werden können, um wirtschaftlichen Zwang auszuüben.

Die Geoökonomik sorgt für eine Verflechtung von Außenwirtschaft(-spolitik) und Außenpolitik (Pisani-Ferry, 2021). Da die EU über eine besondere Kompetenz in der Handelspolitik verfügt, werden zunehmend die begrenzt verfügbaren handelspolitischen Instrumente für eine wachsende Zahl an ökonomischen und politischen Zielen verwendet. Dies war nicht immer so. Handelspolitik hatte ursprünglich das klar definierte ökonomische Ziel der Export- und Importförderung. Weitere Ziele wie die Wohlfahrtsmaximierung und später die Adressierung ökonomischer Ungleichheit führten zu einer breiteren Zielsetzung von Handelspolitik, welche auch die Verteilung der Handelsgewinne adressierte. In den vergangenen Jahren begann die europäische Handelspolitik unter der Einbeziehung von Umwelt-, Menschenrechts-, Arbeits- und Sozialstandards, zu einem Instrument der Außenpolitik zu werden. Dabei wird es immer schwieriger, zwischen den unterschiedlichen Zielen der strategischen Autonomie, Offenheit und Nachhaltigkeit abzuwägen (Kamin et al., 2021).

Welche Möglichkeiten haben also Deutschland und die EU, sich im internationalen Machtspiel zu behaupten und dennoch die Prinzipien der Offenheit und Nachhaltigkeit nicht zu verletzen?

Für die EU ebenso wie für Deutschland ist der Außenhandel eine der wichtigsten Säulen. 14,5 % der weltweiten Exporte (gefolgt von China mit 10,6 % und den USA mit 10,1 %) entfielen im Jahr 2019 auf die EU27. Und auch bei den Importen von Waren und Dienstleistungen lag die EU27 im Jahr 2019 mit rund 13,1 % vor den USA (12,8 %) und China (10,2 %) weltweit in Führung.[1] Die EU hat daher einen starken Anreiz, für offenen und fairen Handel, klar geregelt durch multilaterale Institutionen, einzutreten. Gremien wie die G7 und G20 stellen dafür einen guten Rahmen, und die Wichtigkeit der EU als Einheit gibt ihr eine gute Ausgangsposition zur Durchsetzung ihrer Interessen.

Der Fakt, dass die EU nach wie vor die wichtigste Handelspartnerin für viele Länder ist, ermöglicht ihr beträchtlichen Einfluss auf die Festlegung von internationalen Standards für Waren und Dienstleistungen, da sie im Gegenzug für den Zugang zum EU-Binnenmarkt eine Angleichung an diese Standards von ihren Handelspartnern verlangen kann. Dieser „Brussels Effect" (Bradford, 2020) bedeutet, dass die EU ihre eigenen Vorschriften und Normen extra-territorialisieren und damit ihre Einflusssphäre ausweiten kann. Dies lässt sich z. B. bei der Datenschutz-Grundverordnung und der Chemikalienverordnung REACH sowie in vielen anderen Bereichen beobachten (Gehrke, 2020). Dabei hängt die Stärke des Brussels Effect maßgeblich von der Größe und Qualität des EU-Binnenmarktes ab und hat genau dort auch seine Grenzen. So bestehen Handelshindernisse in der EU aufgrund sprachlicher, kultureller und rechtlicher Unterschiede zwischen den einzelnen EU-Ländern. Diese Heterogenität ist mit höheren Kosten für ausländische Anbieter verbunden.[2]

Vor dem Hintergrund der Verschiebung der globalen wirtschaftlichen und politischen Ordnung im vergangenen Jahrzehnt ist die Ausweitung der bilateralen Handelspartnerschaften der EU von strategischer Bedeutung. Derzeit hat die EU 36 Handelsabkommen mit 77 Handelspartnern abgeschlossen, und etwa 25 Handelsabkommen werden verhandelt. Über Handels- und Investitionsabkommen hat die EU die Möglichkeit, ihre geoökonomische Reichweite zu vergrößern. So verbessert beispielsweise das Ende 2020 mit China verhandelte Comprehensive Agreement on Investment die Verhandlungsposition der EU gegenüber China, indem es die Bedingungen für europäische Unternehmen in Bezug auf fairen Wettbewerb und Marktzugang in China verbessert. Gleichzeitig besteht jedoch die Kritik, dass es immer noch an Investitionsschutzstandards und Investor-Staat-Streitbeilegungsverfahren mangelt und dass beispielsweise die Regeln zur Einhaltung von Umwelt- und Arbeitsstandards zu schwach ausgeprägt seien (Bickenbach und Liu, 2021). Ein Handelsabkommen mit den USA steht noch aus, jedoch hat die Notwendigkeit für eine transatlantische Kooperation seit dem Scheitern der TTIP-Verhandlungen nicht an Wichtigkeit eingebüßt. Solche und weitere Abkommen mit vielen anderen Partnern wie z. B. Indien und Indonesien, aber auch dem Mercosur, sind für die Stärkung der Position der EU elementar, können aber gleichzeitig auch ein wichtiger Schritt hin zur Stärkung multilateraler Institutionen sein.

Natürlich stehen der EU auch Instrumente zur Verfügung, mit denen sie sich im Rahmen des internationalen Handelsrechts durch protektionistische Maßnahmen gegen wettbewerbswidriges Verhalten durch Handelspartner wehren kann. Zum einen gibt es Handelsschutzinstrumente wie Antidumping- sowie Ausgleichs und Schutzzölle. Ein weiteres Instrument sind die oben bereits erwähnten Sanktionen. Die Wirksamkeit von Sanktionen zur Erreichung politischer Ziele ist umstritten, anders als es die negativen wirtschaftlichen Folgen sind, die sie für die Zielländer in der Regel haben. Oftmals trifft dies aber auch auf die sanktionierenden Staaten zu (Chowdhry et al., 2020b).

In der kurzen Frist ist nicht mit einer Umkehr des Trends hin zur Geoökonomie zu rechnen. Ganz im Gegenteil kann vermutet werden, dass sich der Trend im Zuge der erwachenden Machtinteressen der verschiedenen Global Player auch längerfristig verstetigen wird. Ist die Globalisierung also in einer Krise?

Obwohl der Güterhandel besonders von den aktuellen Friktionen wie Brexit, COVID-19 und Lieferkettenengpässen betroffen ist, wird deutlich, dass der Dienstleistungshandel wächst und hier auch noch Potenziale auszuschöpfen sind. Angesichts der vielfältigen adversen geopolitischen Entwicklungen und den damit verbundenen protektionistischen Maßnahmen ist es vielleicht eher verwunderlich, dass der internationale Handel trotz allem noch relativ stark wächst – im Großen und Ganzen gemeinsam mit der globalen Produktion, wie man es erwarten würde. Dies zeigt, dass die Fundamente der Globalisierung trotz allen negativen Einflüssen stark sind. Daher sollte man vielleicht weniger davon sprechen, dass die Globalisierung in einer Krise ist, sondern eher, dass die internationalen Beziehungen auf einem Tiefpunkt sind. Und dies kann leider noch weitreichendere Folgen haben als einen Rückgang im internationalen Handelsvolumen.

1 Eigene Berechnungen auf Basis der Eurostat Database, https://ec.europa.eu/eurostat/data/database/.

2 Ein weiteres Hindernis für den Brussels Effect entsteht dort, wo andere Länder strengere Regulierungen erlassen. Hier müssen EU-Unternehmen sich ausländischen Vorschriften anpassen, um nicht den Marktzugang zu verlieren.

Prof. Holger Görg, Ph. D., leitet das Kiel Centre for Globalization und ist Research Fellow am Kieler Institut für Weltwirtschaft (IfW).

Dr. Katrin Kamin ist stellvertretende Leiterin der Task Force des Präsidenten und leitet in Gemeinschaft die Trade Policy Task Force des Instituts für Weltwirtschaft (IfW).


Literatur

Bickenbach, F. und W.-H. Liu (2021), Das Investitionsabkommen der EU mit China aus europäischer Sicht: Erfolge mit Defizite, Kiel Focus, 02/2021.
Bradford, A. (2020), The Brussels Effect, Oxford University Press.
Chowdhry, S. und G. Felbermayr (2020), The US-China Trade Deal: How the EU and WTO lose from managed trade, Kiel Policy Brief, 132, Kieler Institut für Weltwirtschaft.
Chowdhry, S., A. Jacobs und K. Kamin (2020a), A crisis in times of crisis: Combating COVID-19 under sanctions in Iran, Kiel Policy Brief, 137, Kieler Institut für Weltwirtschaft.
Chowdhry, S., G. Felbermayr, J. Hinz, K. Kamin, A. Jacobs und H. Mahlkow (2020b), The economic cost of war by other means, Kiel Policy Brief, 147, Kieler Institut für Weltwirtschaft.
Felbermayr G Kirilakha A Syropoulos C Yalcin E Yotov Y V The global sanctions data base European Economic Review 2020 129 103561 10.1016/j.euroecorev.2020.103561
Felbermayr, G., K. Kamin, S. Chowdhry, J. Hinz, A. Jacobs, S. Kill und A. Sandkamp (2021), Perspektiven einer erfolgreichen europäischen Handelspolitik im Kontext geopolitischer Herausforderungen. IfW Studie für das Bundesministerium für Digitalisierung und Wirtschaftsstandort.
Flach, L., J. Gröschl, M. Steininger, F. Teti und A. Baur (2021), Internationale Wertschöpfungsketten — Reformbedarf und Möglichkeiten, Ifo-Studie für die Konrad-Adenauer-Stiftung e. V.
Fuchs, A., L. C. Kaplan, K. Kis-Katos, S. Schmidt, F. Turbanisch und F. Wang (2020), Mask wars: China’s exports of medical goods in times of COVID-19, Kieler Arbeitspapiere, 2161, Kieler Institut für Weltwirtschaft.
Garcia-Herrero, A., G. Wolff, J. Xu, N. Poitier, G. Felbermayr, R. Langhammer, W.-H. Liu und A. Sandkamp (2020), EU-China trade and investment relations in challenging times, Europäisches Parlament.
Gehrke, T. (2020), What Could a Geoeconomic EU Look Like in 2020?, EGMONT Security Policy Brief, 123.
Gelpern, A., S. Horn, S. Morris, B. Parks und C. Trebesch (2021), How China Lends: A Rare Look into 100 Debt Contracts with Foreign Governments. Peterson Institute for International Economics, Kiel Institute for the World Economy, Center for Global Development, and AidData at William & Mary.
Kamin, K., K. Bernoth, J. Dombrowski, G. Felbermayr, M. Fratzscher, M. Hoffmann, S. Horn, K. Neuhoff, N. Poitiers, M. Rieth, A. Sandkamp, P. Weil, G. Wolff und G. Zachmann (2021), Instruments of a Strategic Foreign Economic Policy, IfW Studie für das Auswärtige Amt.
Mao H Görg H Friends like this: The impact of the US — China Trade War on Global Value Chains The World Economy 2020 43 7 1776 1791 10.1111/twec.12967
Pisani-Ferry, J. (2021), The Geopolitical Conquest of Economics, Project Syndicate.
